# System analysis of gene mutations and clinical phenotype in Chinese patients with autosomal-dominant polycystic kidney disease

**DOI:** 10.1038/srep35945

**Published:** 2016-10-26

**Authors:** Meiling Jin, Yuansheng Xie, Zhiqiang Chen, Yujie Liao, Zuoxiang Li, Panpan Hu, Yan Qi, Zhiwei Yin, Qinggang Li, Ping Fu, Xiangmei Chen

**Affiliations:** 1 Department of Nephrology, Chinese PLA General Hospital, Chinese PLA Institute of Nephrology, State Key Laboratory of Kidney Diseases, National Clinical Research Center for Kidney Diseases, Fuxing Road 28, Beijing, 100853, P.R. China.; 2 Department of Nephrology, Beijing Chao-Yang Hospital, Beijing, China; 3 Medical College, Nankai University, Tianjin, China; 4 BGI-Shenzhen, Shenzhen, China; 5 Department of Nephrology, West China Hospital of Sichuan University, Chengdu, Sichuan, China; 6 Department of Nephrology, Civil Aviation General Hospital, Beijing, China; 7 Department of Nephrology, Tianjin Medical University General Hospital, Tianjin, China

## Abstract

Autosomal dominant polycystic kidney disease (ADPKD) is the most common inherited kidney disorder mainly caused by mutation in PKD1/PKD2. However, ethnic differences in mutations, the association between mutation genotype/clinical phenotype, and the clinical applicable value of mutation detection are poorly understood. We made systematically analysis of Chinese ADPKD patients based on a next-generation sequencing platform. Among 148 ADPKD patients enrolled, 108 mutations were detected in 127 patients (85.8%). Compared with mutations in Caucasian published previously, the PKD2 mutation detection rate was lower, and patients carrying the PKD2 mutation invariably carried the PKD1 mutation. The definite pathogenic mutation detection rate was lower, whereas the multiple mutations detection rate was higher in Chinese patients. Then, we correlated PKD1/PKD2 mutation data and clinical data: patients with mutation exhibited a more severe phenotype; patients with >1 mutations exhibited a more severe phenotype; patients with pathogenic mutations exhibited a more severe phenotype. Thus, the PKD1/PKD2 mutation status differed by ethnicity, and the PKD1/PKD2 genotype may affect the clinical phenotype of ADPKD. Furthermore, it makes sense to detect PKD1/PKD2 mutation status for early diagnosis and prognosis, perhaps as early as the embryo/zygote stage, to facilitate early clinical intervention and family planning.

Autosomal dominant polycystic kidney disease (ADPKD) is the most common inherited kidney disorder with a 50% risk of inheritance^1^. Approximately 50% of ADPKD patients progress to end-stage renal disease (ESRD) before age 60^2^,^3^, making ADPKD the fourth leading cause of ESRD that heavily burdens social and families^4^. Therefore, delaying the progression and reducing the incidence of ADPKD are important from both a research and clinical perspective.

ADPKD is genetically heterogeneous, and two genes, PKD1 and PKD2, have been identified to participate in this disease^5^. Previous studies of PKD1/PKD2 mutations mainly focused on Caucasians, and this mutation has not been thoroughly analysed in large samples of the Asian population. Furthermore, differences between Caucasian and Asian populations are poorly understood.

ADPKD is a chronic progressive disease that is mainly diagnosed by renal imaging techniques coupled with an age-specific renal phenotype^6^,^7^, and effective clinical treatments for this disease are currently lacking. Therefore, the early diagnosis of ADPKD using genetic testing prior to clinical imaging diagnosis, the appropriate monitoring of clinical indexes and timely symptomatic treatment may delay the progression of ADPKD. Notably, reducing the incidence of new cases by detecting disease-causing gene mutations in embryos or zygotes of patients with ADPKD and providing reasonable fertility recommendations may reduce the incidence of this disease. Although correlations between the phenotype and genotype in ADPKD patients have been reported in previous studies, the correlation between the genotype (such as with/without mutation, mutation number, mutation position, and mutation type) and clinical phenotype has not yet been described in detail. Therefore, detecting mutations in ADPKD patients may not only provide evidence for ADPKD diagnosis but also provide reference information to predict ADPKD progression and permit family planning. To this end, sequencing technology has rapidly developed in recent years. Specifically, next-generation sequencing (NGS) has been widely used to study gene testing for genetic diseases due to its advantages of high coverage and deep sequencing as well as its ability to simultaneously analyse several samples^8^. Therefore, NGS may be used to detect ADPKD mutations to broaden the use of genetic diagnosis in the setting of ADPKD.

This study aimed to systematically analyse Chinese ADPKD patients based on a NGS platform. Specifically, ① we detected mutations in the target region (PKD1 and PKD2) in Chinese patients and compared the resultant data with mutations previously detected in Caucasian patients; ② we systematically associated mutations in PKD1/PKD2 and clinical data.

## Results

### Patient characteristics

One hundred and forty-eight patients with ADPKD were enrolled in this study. The male to female ratio was 70:78, and the mean age of patients was 43.47 ± 12.73 years. The mean age at diagnosis was 34.08 ± 10.07 years (range, 12–66 years). Eighty-two patients (55.4%) had clear family history.

### Description of mutations in targeted region

**Figure 1:**
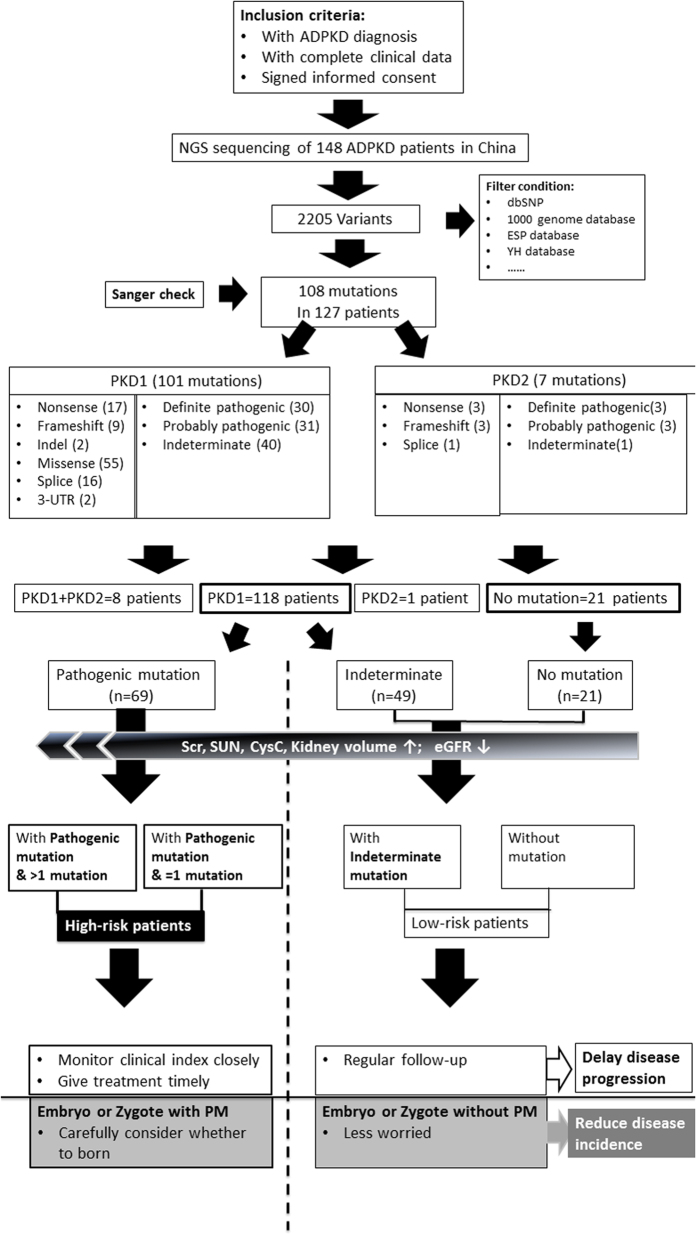
Flow diagram of genetic diagnosis (mutation detection and pathogenic prediction in PKD1/PKD2) based on the next-generation sequencing platform, and the clinical significance of genetic diagnosis for delaying progression and reducing the incidence of ADPKD: ① one hundred and forty eight patients diagnosed with ADPKD were enrolled, and their peripheral blood was subjected to next-generation sequencing. After a comparison with databases, normal variations and artefact variants were filtered out, resulting to 108 mutations detected in 127 patients (85.8%). Of these 148 patients, one hundred and eighteen (79.7%, 118/148) harboured a mutation in PKD1, 1 (0.7%, 1/148) harboured a mutation in PKD2, 8 (5.4%, 8/148) harboured mutations in both PKD1 and PKD2, and 21 lacked PKD1/PKD2 mutations (14.2%, 21/148). The pathogenicity of mutations was predicted, and they were categorized into three types (definite pathogenic mutation, probable pathogenic mutation, and indeterminate mutation). Thus, the patients were divided into three groups: patients with pathogenic mutation, patients with indeterminate mutation, and mutation-free patients. The association of genotype/phenotype showed that patients with a pathogenic mutation had higher serum creatinine levels, higher serum urea nitrogen levels, higher cystatin c levels, larger kidney volumes, and lower eGFR levels than patients with indeterminate mutations or mutation-free patients. Based on these data, ADPKD patients were categorized into two groups: the high-risk group (with pathogenic mutations) and the low-risk group (with indeterminate mutation or no mutation). ② Including genetic diagnosis in clinical practice will likely reduce the economic cost of the disease and reduce monitoring patients destined to be symptom-free while proactively increasing preventive monitoring for patients at high risk for progressive renal disease to help delay the progression of ADPKD. In addition, genetic diagnoses of embryos or even zygotes for ADPKD patients who have a family plan may provide reasonable fertility recommendations to help decrease the incidence of ADPKD.

The quality of NGS data were shown in Supplementary Table 1. A total of 108 mutations were identified (101 and 7 mutations found in PKD1 and PKD2, respectively) (Supplementary Table 2). The *novel* mutation detection rate was 70.4% (76/108). Thirty-five *novel* mutations without clear family history were identified among the total 148 ADPKD patients. The pathogenic predictions were shown in Fig. 1 and Supplementary Table 3.

In our enrolled cohort, 21 patients did not harbour mutations in either the PKD1 or PKD2 gene (14.2%, 21/148). One hundred-eighteen (79.7%, 118/148) harboured PKD1 mutation, 1 (0.7%, 1/148) harboured PKD2 mutation, and 8 (5.4%, 8/148) harboured mutations in both PKD1 and PKD2; the mutation detection rate was 85.8% (127/148). Seventy one (48.0%, 71/148) patients had at least two mutations in the targeted region. The definite pathogenic mutation detection rate was 27.7% (41/148), the probable pathogenic mutation detection rate was 23.6% (35/148).

### Comparison of mutation features of PKD1/PKD2 between Caucasian and Chinese patients

As shown in Table 1, a literature review yielded 21 studies (13 on Caucasian, 8 on Asian) in which PKD1/PKD2 was entirely screened for mutation detection. This study examined the largest sample of Chinese ADPKD patients to date. First, we compared the mutation rates between Caucasian and Chinese patients: ① the mutation detection rate in reached ~80% in most studies of Caucasian patients (ranging from 62.8% to 89.9%), and the mutation detection rate was 85.8% in this study; ② the majority of studies of Caucasian populations reported a PKD2 mutation detection rate ranging from 10.3–17.4%, and the proportion of PKD1 and PKD2 mutations were approximately 85% and 15%, respectively, in Caucasians, whereas the proportion of PKD2 mutations was less than 6.1% (including patients who also harboured a PKD1 mutation) in this study; ③ADPKD patients were divided into PKD1 patients or PKD2 patients based on the independent presence of PKD1/PKD2 in Caucasian populations, but eight of the 9 patients harbouring a PKD2 mutation also harboured a PKD1 mutation in this study; in other words, only one patient harboured only a PKD2 mutation in this study. In addition, Chinese ADPKD patients in this study were a similar age or even older than Caucasian patients; ④ most Caucasian patients harboured only single unique mutations, whereas almost half of all ADPKD patients (48.0%) in this study harboured more than one mutation; ⑤ the definite pathogenic mutation detection rate was lower in Chinese individuals (27.7%) than in Caucasian individuals (ranging from 35.1% to 66.6%).

Of the 8 studies of Asian populations, only one enrolled more than 100 patients (Japanese); therefore, we also compared mutations between Chinese and Japanese individuals. We found that the proportion of PKD2 mutations in Japanese patients (28.1%) was higher than that in Chinese individuals and in Caucasian individuals, and the definite pathogenic mutation detection rate in Japanese individuals (52.2%) was higher than that in Chinese individuals.

### Relationship between genotype and phenotype

#### Influence of with/without mutation detected targeted region on phenotype

As shown in Table 2, the serum creatinine, serum urea nitrogen, and CysC levels were significantly higher in patients with mutations (*P* = 0.003, 0.013, and 0.009, respectively) than in mutation-free patients. The level of eGFR was lower (*P* = 0.005) and the kidney volume was larger (*P* = 0.011) in patients with mutations than in mutation-free patients.

#### Influence of PKD2 mutation on phenotype

As shown in Supplementary Table 4, we made comperasion between patients harboring indeterminate PKD1 mutation & PKD2 mutation and patients harboring indeterminate PKD1 mutation, then we only found the differences on in incidence of polycystic liver and serum total protein and there was no differences on kidney phenotype.

#### Influence of mutation number on phenotype

Because only 9 patients of the enrolled patients harboured a PKD2 mutation, they were removed from the subsequent analysis of the association between the PKD1 mutation rate and clinical phenotype. We compared patients with one mutation to those with more than one mutation and found that the kidney volume was significantly larger (*P* = 0.035) and eGFR expression was significantly lower (*P* = 0.033) in patients with more than one mutation than in patients with one mutation (Table 3).

#### Influence of mutation position on phenotype

We evaluated the influence of mutation location along the PKD1 gene; specifically, patients with one mutation in the PKD1 gene were separated into three groups according to the mutation position (group 1, 5′ end to position 2147228; group 2, position 2150076–2156600; group 3, position 2158432 to 3′), and we did not find significant differences in the clinical data (Supplementary Table 5).

#### Influence of mutation pathogenic type on phenotype

To eliminate the effect of multiple mutations on phenotype, we analysed patients with a single PKD1 mutation (n = 55). Compared with patients with a definite pathogenic mutation and those with a probable pathogenic mutation, patients with an indeterminate mutation exhibited a better phenotype (Supplementary Table 6). Because patients with definite and probable pathogenic mutations did not significantly differ, we combined these patients into one group (pathogenic mutation group). Compared with patients with an indeterminate mutation and patients without mutation, patients with a pathogenic mutation exhibited a poorer phenotype (Supplementary Table 7). Although the clinical data did not significantly differ between patients with an indeterminate mutation and those without mutations, patients with indeterminate mutations tended to have higher serum creatinine, higher serum urea nitrogen, higher CysC, and lower eGFR levels.

We then divided patients who harboured PKD1 mutation or were free of mutations into three groups, i.e., patients with pathogenic mutation, patients with indeterminate mutation, and patients without mutations, and compared these groups. As shown in Table 4, patients with pathogenic mutations had higher serum creatinine levels (*P* < 0.001), serum urea nitrogen levels (*P* = 0.008, 0.002), CysC levels (*P* = 0.001, 0.002) and kidney volumes (*P* = 0.021, 0.004) and lower eGFR levels (*P* < 0.001) than patients harbouring indeterminate mutations and mutation-free patients. The clinical data of patients with indeterminate mutations and those without mutations did not significantly differ.

## Discussion

This study features 3 innovative aspects: ① we screened PKD1/PKD2 mutations in the largest sample of Chinese ADPKD patients using an advanced sequencing technique (targeted next-generation sequencing); ② this study was the first to compare mutation features between Chinese (Asian) patients and Caucasian (Western) patients with ADPKD; ③ we correlated the detailed genotype (with/without mutation, mutation number, mutation location, mutation pathogenic type etc.) and clinical phenotype (detailed clinical data).

In the present study, one hundred eight mutations were detected using NGS, all of which were confirmed by Sanger sequencing, which demonstrates that the targeted NGS platform was reliable to detect PKD1 and PKD2 mutation. Thus, the targeted NGS platform reduced the time and cost necessary for the genetic diagnosis of ADPKD and may serve as an efficient approach to detect mutations in PKD1/PKD2.

Based on the targeted NGS platform, we the analysed genotype and phenotypes of ADPKD patients in China. First, the comparison of Chinese and Caucasian patients yielded the following five findings: ① in this study, the overall detection rate was 85.8% and agreed with the detection levels of 89.1% and 89.9% in Caucasians reported by Rossetti *et al*.^9^ and Audrezet *et al*.^10^ in other words, the detection rate remained consistent between Chinese and Caucasian individuals; ② the majority of studies of Caucasian patients reported a PKD2 mutation detection rate ranging from 10.3%-17.4%, whereas only one patient harboured a single PKD2 mutation in this study, suggesting that the PKD2 detection rate might be higher in Caucasians than in Chinese individuals; ③ the majority of Caucasian patients harboured a unique mutation in PKD1 or PKD2, whereas 8 of the 9 patients with a PKD2 mutation in this study also had PKD1 mutation; therefore, we speculated that PKD2 mutations may accompany PKD1 mutations more commonly in Chinese patients. Then we compared the age between Caucasian and Chinese patients, and found that compared with Carcasian patients, Chinese ADPKD patients in this study were a similar age or even older, which might be a more evidence for that the incidence of PKD2 mutation in Chinese is lower. ④ among the 148 patients in this study, seventy one patients (48.0%) harboured more than one mutations. However, no single mutation accounted for >2% of all unrelated ADPKD patients in previous studies (Caucasian population)^10^. Thus, patients with more than one mutation were more common in Chinese; ⑤ we found that the definite pathogenic mutation detection rate in this study was lower than those reported in Caucasian patients. Thus, the PKD1/PKD2 mutation rate may differ by ethnic group, which may explain the epidemiology of ADPKD. In addition, we found differences in the PKD2 mutation proportion and definite pathogenic mutation detection rate between Chinese and Japanese individuals, which may be due to differences among Asian populations or differences in the sequencing methods between the two studies. Thus, further studies utilizing different arrays are warranted.

Then, we correlated the mutation characteristic (genotype) in PKD1/PKD2 and clinical phenotype in detail. This analysis yielded the following findings ① mutation-free patients exhibited a milder phenotype than patients harbouring a mutation; ② the ADPKD patients harboring both PKD1 and PKD2 mutation did not have a more severe clinical phenotype than the patients only harboring PKD1 mutation; ③ patients with more than one mutation exhibited a poorer phenotype than patients harbouring a single mutation; ④ mutation location may not affect the clinical phenotype; ⑤ patients harbouring a pathogenic mutation (definite pathogenic mutation or probable pathogenic mutation) exhibited poorer renal function than patients with an indeterminate mutation and mutation-free patients. These findings suggested that the presence, frequency, and pathogenic type of PKD1 might significantly affect the clinical phenotype. Thus, multiple mutations and at least one pathogenic mutation would predict a severe clinical manifestation and poor prognosis. Consequently, mutation sequencing information may not only diagnose but also predict the prognosis of ADPKD, which facilitates personalized patient management and provides family planning advice. Therefore, we should closely monitor the clinical indexes and administer timely treatment to high-risk patients (with multiple mutation/pathogenic mutation), whereas only regular follow-up is necessary for low-risk patients (with indeterminate mutations or no mutation). These interventions may help delay the progression of ADPKD. In addition, a genetic diagnosis based on mutation detection in embryos or even zygotes and defining mutation number and pathogenic type for ADPKD patients undergoing family planning may provide reasonable fertility recommendations to help decrease the incidence of ADPKD. Given the reduced cost of gene sequencing, such as targeted NGS, and the increasing number ADPKD patients whose phenotype is known, bringing PKD mutation types to clinical practice will likely reduce the economic cost of disease and reduce monitoring in patients destined to be symptom free while proactively increasing preventive monitoring for patients at high risk for progressive renal disease. In addition, defining the genetic mutation in ADPKD will better define the appropriate patient population for randomized clinical trials and develop new rationales for treatment using the molecular information obtained from locus and mutation detection^8^,^10^,^11^,^12^,^13^,^14^,^15^,^16^,^17^,^18^,^19^,^20^,^21^,^22^,^23^,^24^,^25^,^26^,^27^,^28^.

In conclusion, NGS may be an optimal sequencing technology to detect causative mutations in ADPKD patients because it increases the mutation detection rate while reducing test costs and turnaround time. As shown in Fig. 1, the clinical phenotype is related to the genotype (with/without mutation, mutation number, and mutation type) in the targeted region. Therefore, mutation sequencing of the targeted gene (PKD1/PKD2) should not be limited to the diagnosis of ADPKD but is of great significance as predictor of prognosis for ADPKD patients. Thus, this approach may help physicians and patients to take optimal measures to delay the progression and decrease the incidence of ADPKD, which is highly important for the long-term prevention of ADPKD (Fig. 1).

## Materials and Methods

### Patients

Patients who were outpatients/inpatients at the Chinese PLA General Hospital in Beijing or West China Hospital in Chengdu City Sichuan Province between April 2012 and March 2014, and diagnosed with ADPKD were enrolled in this study. The ADPKD diagnosis was based on kidney ultrasound findings in accordance with previously described criteria^29^.

Approximately 5 ml of peripheral blood was obtained from the participants using an EDTA anticoagulation tube and sodium citrate anticoagulation tube. The DNA was extracted from leukocytes using standard methods and stored at −80 °C.

### Ethics statement

This study adhered to the Declaration of Helsinki and has been reviewed and approved by the Ethics Committee of the Chinese PLA General Hospital (Ethics Approval Number: No. 2012-001). Informed consent was obtained from all of enrolled individuals.

### Targeted next-generation sequencing, mutation identification and pathogenic conformation

The detailed methods were shown in supplementary materials (Supplemental Fig. 1).

### Literature search and screen

A comprehensive literature search for studies published until August 2015 was undertaken in PUBMED using the following relevant index words: “autosomal dominant polycystic kidney disease/polycystic kidney disease/ADPKD” and “mutation/sequencing/PKD1/PKD2”. We also checked the references of the included trials to identify other studies. The studies in which PKD1 was not entirely screened were excluded.

### Statistical analyses

All statistical analyses were performed using the SPSS software, version 19 (SPSS, Inc., Armonk, NY). Normally distributed continuous variables are expressed as the mean ± SD, and non-normally distributed variables are expressed as the median and interquartile range (IQR). The normality of continuous variables was visually assessed using Q-Q plots and the Kolmogorov-Smirnov test. Groups were compared using the unpaired *t*-test, chi-squared test, or Mann-Whitney/Kruskal-Wallis test. *P* < 0.05 was considered to indicate significant differences^13^,^30^,^31^.

## Additional Information

**How to cite this article**: Jin, M. *et al*. System analysis of gene mutations and clinical phenotype in Chinese patients with autosomal-dominant polycystic kidney disease. *Sci. Rep.* **6**, 35945; doi: 10.1038/srep35945 (2016).

**Publisher’s note:** Springer Nature remains neutral with regard to jurisdictional claims in published maps and institutional affiliations.

## Supplementary Material

Supplementary Information

## Figures and Tables

**Table 1:** Summarizing published studies performing entire screening for PKD1/PKD2 mutation detection.

Publish year	Ethnicity	No. of patients/family	Age	Gene detected	Sequencing Method	Mutation detection rate (no. patients/familial)	Mutation recurrence rate	Multiple mutation rate	PKD1	PKD2	Definite mutation rate in patient %	Reference
*Caucasian*												
2002 (Burtey *et al*.)	French	9	—	PKD1	RT-PCR, DS	66.7% (6)	0	0	—	—		12
2002 (Rossetti *et al*.)	American	45	—	PKD1 PKD2	DHPLC, DS	77.8% (35)	2.9 (1/34)	0	91.4 (32/35)	8.6 (3/35)	64.4 (29/45)	30
2005 (Peltola *et al*.)	Finnish	17	43 ± 2	PKD1 PKD2	LR-PCR, DS, SSCP	100% (17)	0	0	94.1 (16/17)	5.9 (1/17)	64.7	13
2007 (Rossetti *et al*.)	American	202	15-46	PKD1 PKD2	DHPLC, LR-PCR, DS	89.1% (180)	30.0		85 (153/180)	15 (27/180)	62.9 (127/202)	9
2007 (Garcia-Gonzalez *et al*.)	Canadian	82	46.5 (1-73)	PKD1 PKD2	LR-PCR, DS	78.0% (64)	0	0	79.7 (51/64)	20.3 (13/64)	41.5	14
2008 (Tan *et al*.)	American	22	—	PKD1 PKD2	LR-PCR, SURVEYOR Nuclease, WAVE Nucleic Acid High Sensitivity Fragment Analysis System	86.3% (19)	0	0	84.4	15.6	63.6 (14/22)	15
2011 (Bataille *et al*.)	French	37	51 ± 11	PKD1 PKD2	LR-PCR, RT-PCR, HRM, DS	75.7% (28)	0	0	89.3 (25/28)	10.7 (3/28)	35.1	16
2011 (Hoefele *et al*.)	German	93	—	PKD1 PKD2	LR-PCR, DS	64.5% (60)	0	0	86.7	13.3		17
2012 (Rossetti *et al*.)	American	183	—	PKD1 PKD2	Next-generation sequencing	62.8% (115)		12.6 (23/183)	82.6	17.4	36.1 (66/183)	18
2012 (Audrezet *et al*.)	French	700	—	PKD1 PKD2	DS, QFM-PCR, array-CHG	89.9% (629)	20.8 (92/442)		83.8 (527/629)	16.2 (	66.6	10
2013 (Neumann *et al*.)	South-western German	277	—	PKD1 PKD2		64.6% (179)	16.7 (21/126)	0	81.0 (145/179)	19.0 (34/179)	45.1 (125/277)	19
2014 (Trujillano *et al*.)	Spanish	48	—	PKD1 PKD2	Targeted NGS	93.8 (45/48)	0	2.1 (1/48)	88.9 (40/45)	11.1 (5/45)	64.6 (31/48)	20
2014 (Obeidova *et al*.)	Czech	56	—	PKD1 PKD2	LR-PCR, HRM analysis, MLPA	71.4% (40)	5.1 (2/39)	1.8 (1/56)	95.0 (38/40)	5.0 (2/40)	~46.4 (26/56)	21
*Asian*												
2000 (PHAKDEEKITCHAROEN *et al*.)	Thai and Korean	47 (41 Thai& 6 Korean)		PKD1	LR-PCR, SSCA	34.0 (16/47)	0	0	—	—	19.1 (9/47)	22
2002 (Inoue *et al*.)	Japanese	8 unrelated		PKD1	LR-PCR, DS	87.5%; \	0	12.5 (1/8)	—	—	75 (6/8)	23
2004 (Zhang *et al*.)	Chinese	24		PKD1 PKD2	LR-PCR, DS, SSCP	70.8% (17)	0	0	70.6 (12/17)	29.4 (5/17)	33.3 (8/24)	24
2011 (Yu *et al*.)	Chinese	65		PKD1 PKD2	DHPLC, LR-PCR, DS	52.3% (34)	13.8 (4/29)	0	88.2 (30/34)	11.8 (4/34)	43.1 (28/65)	25
2013 (Chang *et al*.)	Chinese-Taiwanese	46		PKD1 PKD2	LR-PCR, DS, RT-QPCR, MLPA	65.2% (30)	8.8 (3/34)	6.5 (3/46)	76.7 (23/30)	23.3 (7/30)	37.0 (17/46)	26
2014 (Choi *et al*.)	Korean	20		PKD1 PKD2	LR-PCR, DS, MLPA	90% (18)	0	0	83.3 (15/18)	16.7 (3/18)	65.0 (13/20)	28
2014 (Yang *et al*.)	Chinese	7		PKD1 PKD2	Targeted NSG	85.7 (6/7)	0	0			57.1 (4/7)	8
2015 (Kurashige *et al*.)	Japanese	161		PKD1 PKD2	LR-PCR, DS	83.9% (135)	13.0 (14/108)	6.1 (9/148)	71.9 (97/135)	28.1 (38/135)	52.2 (84/135)	27
2015	Chinese	148		PKD1 PKD2	Target NSG	85.8%	28.7 (31/108)	48.0 (71/148)	92.9 (118/127) 99.2 (126/127)	0.8 (1/127) 7.1 (9/127)	27.7 (41/148)	This study

Abbreviations: LR-PCR, long range-PCR; SSCA: single-stand conformation analysis; DS, direct sequencing; DHPLC, denaturing high-performance liquid chromatography; SSCP, single-strand conformation polymorphism; HRM: high resolution melt analysis; QFM-PCR, quantitative fluorescent multiplex PCR; aaray-CGH, array-comparative genomic hybridization; NGS, next-generation sequencing; DP, definitely pathogenic; HLP, highly likely pathogenic; LP, likely pathogenic.

**Table 2:** Influence of presence or absence of mutation in targeted region on clinical phenotype.

Characteristic	With mutation (n = 127)	Without mutation (n = 21)	*P* Value
Sex (male/female)	59/68	11/10	0.644
Age (yr)	44.22 ± 12.80	40.24 ± 11.58	0.185
Age at diagnosis (yr)	34.38 ± 9.80	32.55 ± 12.54	0.573
Clear family history	74 (58.4%)	8 (38.1%)	0.085
Polycystic liver	54 (42.5%)	5 (23.8%)	0.105
hypertension	65 (51.2%)	1 (52.4%)	0.919
Urologic complication	61 (48.0%)	9 (42.9%0	0.660
Born as the first child	98 (77.2%)	18 (85.7%)	0.378
BMI (kg/m^2^)	22.40 (20.96–24.80)	23.09 (20.38–24.85)	0.683
Hemoglobin (g/L)	127.61 ± 22.06	135.09 ± 10.94	0.274
White blood cell count (*10^9^/L)	6.27 ± 2.11	5.93 ± 1.05	0.607
Platelet (*10^9^/L)	207.18 ± 69.64	192.20 ± 59.05	0.519
Serum albumin (g/L)	43.04 ± 5.57	46.42 ± 5.28	0.089
Serum total protein (g/L)	70.72 ± 6.05	74.66 ± 3.65	0.061
Serum creatinine (μmol/L)	103.08 (74.15–205.11)	57.55 (53.50–101.55)	0.003^a^
Serum urea nitrogen (mmol/L)	7.29 (5.45–9.60)	5.03 (3.82–6.99)	0.013^a^
Serum uric acid (μmol/L)	343.97 ± 108.80	303.17 ± 133.22	0.223
CysC (mg/L)	1.12 (0.81–1.86)	0.66 (0.53–1.23)	0.009^a^
eGFR (ml/min)	63.71 (25.65–96.35)	102.72 (72.80–126.90)	0.005^a^
Urine protein quantity (g/24 h)	0.19 (0.06–0.70)	0.15 (0.00–0.60)	0.640
Kidney volume (mm^3^)	754.88 (240.08–1125.54)	188.19 (113.68–353.69)	0.011^a^

BMI, Body Mass Index; eGFR, estimated Glomerular Filtration Rate.

^a^*P* < 0.05 compared with group with mutation.

**Table 3:** Influence of mutation number of PKD1 on clinical phenotype.

Characteristic	Mutation number = 1 (n = 55)	Mutation number > 1 (n = 63)	*P* Value
Sex (male/female)	23/32	32/31	0.330
Age (yr)	42.67 ± 12.91	44.21 ± 12.74	0.539
Age at diagnosis (yr)	33.47 ± 7.46	33.78 ± 11.25	0.889
Clear family history	27 (49.1%)	41 (65.1%)	0.080
Polycystic liver	15 (27.3%)	32 (50.8%)	0.009
hypertension	29 (53.7%)	31 (48.4%)	0.569
Urologic complication	27 (49.1%)	28 (44.4%)	0.614
Born as the first child	6 (10.9%)	18 (28.6%)	0.017
BMI (kg/m^2^)	22.21 (21.22–24.80)	22.12 (2.019–24.80)	0.591
Hemoglobin (g/L)	128.28 ± 19.20	127.97 ± 23.48	0.953
White blood cell count (*10^9^/L)	6.32 ± 1.82	6.15 ± 2.28	0.751
Platelet (*10^9^/L)	210.67 ± 78.38	208.50 ± 66.26	0.903
Serum albumin (g/L)	44.38 ± 4.63	42.46 ± 6.00	0.170
Serum total protein (g/L)	71.29 ± 6.09	71.46 ± 5.84	0.918
Serum creatinine (μmol/L)	95.20 (63.28–193.41)	112.00 (91.05–221.68)	0.035
Serum urea nitrogen (mmol/L)	7.48 (4.99–10.00)	7.06 (5.84–8.62)	0.949
Serum uric acid (μmol/L)	329.47 ± 121.03	365.90 ± 98.07	0.146
CysC (mg/L)	1.02 (0.71–1.78)	1.11 (0.89–2.99)	0.148
eGFR (ml/min)	71.79 (27.09–112.55)	54.51 (25.19–82.97)^a^	0.033
Urine protein quantity (g/24 h)	0.19 (0.050.83)	0.20 (0.06–0.70)	0.693
Kidney volume (mm^3^)	351.97 (199.35–904.72)	864.11 (632.63–1486.92)^a^	0.035

BMI, Body Mass Index; eGFR, estimated Glomerular Filtration Rate.

^a^*P* < 0.05 compared with group with one mutation.

**Table 4:** Influence of mutation pathogenic type of PKD1 on clinical phenotype.

Characteristic	Pathogenic (n = 69)	Indeterminate (n = 49)	Without variants (n = 21)	*P* Value
Sex (male/female)	26/43	29/20	11/10	0.048
Age (yr)	42.31 ± 11.62	44.98 ± 14.06	40.24 ± 11.58	0.263
Age at diagnosis (yr)	30.98 ± 7.43	37.14 ± 11.22	32.55 ± 12.54	0.020
Clear family history	42 (60.9%)	25 (52.1%)	8 (38.1%)	0.172
Polycystic liver	31 (44.9%)	16 (33.3%)	5 (23.8%)	0.161
hypertension	33 (47.8%)	27 (57.4%)	1 (52.4%)	0.595
Urologic complication	27 (39.1%)	28 (58.3%)	9 (42.9%)	0.115
Born as the first child	53 (76.8%)	40 (83.3%0	18 (85.7%)	0.548
BMI (kg/m^2^)	21.88 (20.53–24.76)	22.40 (21.22–25.71)	23.09 (20.38–24.85)	0.581
Hemoglobin (g/L)	127.54 ± 22.91	128.87 ± 19.75	135.09 ± 10.94	0.565
White blood cell count (*10^9^/L)	5.98 ± 1.97	6.54 ± 2.18	5.93 ± 1.05	0.423
Platelet (*10^9^/L)	196.95 ± 66.85	225.48 ± 75.07	192.20 ± 59.05	0.188
Serum albumin (g/L)	42.99 ± 5.64	43.63 ± 5.42	46.42 ± 5.28	0.260
Serum total protein (g/L)	70.59 ± 5.90	72.34 ± 5.85	74.66 ± 3.65	0.150
Serum creatinine (μmol/L)	151.80 (91.15–314.25)	90.30 (64.65–115.50)^a^	57.55 (53.50–101.55)^a^	<0.001
Serum urea nitrogen (mmol/L)	7.92 (6.25–14.05)	7.00 (4.97–8.07)^a^	5.03 (3.82–6.99)^a^	0.001
Serum uric acid (μmol/L)	364.99 ± 139.47	323.35 ± 111.34	303.17 ± 133.22	0.142
CysC (mg/L)	1.44 (0.89–3.71)	0.92 (0.71–1.37)^a^	0.66 (0.53–1.23)^a^	<0.001
GFR (ml/min)	39.02 (18.81–83.89)	79.13 (55.85–108.75)^a^	102.72 (72.80–126.90)^a^	<0.001
Urine protein quantity (g/24 h)	0.19 (0.07–0.70)	0.19 (0.00–1.43)	0.15 (0.00–0.60)	0.845
Kidney volume (mm^3^)	846.64 (421.27–1217.30)	269.61 (193.17–742.87)^a^	188.19 (113.68–353.69)^a^	0.004

BMI, Body Mass Index; eGFR, estimated Glomerular Filtration Rate.

^a^*P* < 0.05 compared with group with pathogenic mutation.
