# Supplementary material for: System analysis of gene mutations and clinical phenotype in Chinese patients with autosomal-dominant polycystic kidney disease
Source: Sci Rep. 2016 Oct 26;6:35945. doi: 10.1038/srep35945 (PMC5080601; doi:10.1038/srep35945)
Supplement: Supplementary Information [file srep35945-s1.pdf]

System analysis of gene mutations and clinical phenotype in Chinese patients with autosomal-dominant polycystic kidney disease

Meiling Jin, MS[1,2,#], Yuansheng Xie, MD, PhD[1,#*], Zhiqiang Chen, MS[3], Yujie Liao, MD, PhD[4], Zuoxiang Li, MS[1], Panpan Hu, MS[1,5,], Yan Qi, MB[1], Zhiwei Yin, PhD[1], Qinggang Li, MD, PhD[1], Ping Fu, MD[4], Xiangmei Chen, MD, PhD[1,*]

[1] Department of Nephrology, Chinese PLA General Hospital, Chinese PLA Institute of Nephrology,

State Key Laboratory of Kidney Diseases, National Clinical Research Center for Kidney Diseases,

[2] Medical College, Nankai University, Tianjin, China

[3] BGI-Shenzhen, Shenzhen, China

[4] Department of Nephrology, West China Hospital of Sichuan University, Chengdu, Sichuan, China.

[5] Department of Nephrology, Civil Aviation General Hospital, Beijing, China

[#] Contributed equally

[*] Co-Corresponding author

Supplementary Methods

Targeted next-generation sequencing and mutation identification

Approximately 5 ml of peripheral blood was obtained from the participants using an EDTA anticoagulation tube and sodium citrate anticoagulation tube. The DNA was extracted from leukocytes using standard methods and stored at -80 °C.

A custom capture array (NimbleGen, Roche) was designed to capture the exons, splice sites

and the immediately adjacent intron sequences of targeted genes (PKD1 and PKD2). The qualified genomic DNA sample was then fragmented using AFA (Adaptive Focused Acoustics™) technology on Covaris S220 Focused-ultrasonicators, and the sizes of the library fragments were primarily distributed between 250 bp and 300 bp. Adapters were then ligated to both ends of the resulting fragments. Extracted DNA was then amplified by ligation-mediated PCR (LM-PCR), purified, and hybridized to the Nimblegen SeqCap EZ Library for enrichment; non-hybridized fragments were then washed out. Both non-captured and captured LM-PCR products were subjected to quantitative PCR to estimate the magnitude of enrichment. Each captured library was then loaded on a Hiseq2000 platform, and we performed high-throughput sequencing for each captured library to ensure that each sample met the desired average sequencing depth. Raw image files were processed using the Illumina basecalling Software 1.7 for base-calling with default parameters, and the sequences of each individual were generated as 90 bp pair-end reads. SOAPaligner/SOAP2 was used to map reads onto the reference, whereas BWA was used to map reads to the reference when analysing indels. The information of BWA can be found at http://bio-bwa.sourceforge.net/. The variations identified with NGS were validated by using classic Sanger sequencing. The standard nomenclature recommended by HGVS (http://www.hgvs.org/mutnomen/) was used to name the variations in this study(1).

Common polymorphisms were filtered out by comparison with databases. We used dbSNP 137, the 1000 genome database, the 1000 genome (1092) database, the Exon Sequencing Project database (http://evs.gs.washington.edu), an in-house exome variant dataset, the HapMap database, the PVFD database (variant frequency in normal population database), and YINXIE and YH databases, which are based on the Chinese population, to filter out both common benign variants

and recurrent artefact variant calls. All detected variants were compared to gene mutation databases (The Human Gene Mutation Database [HGMD], http://www.hgmd.cf.ac.uk and ADPKD Database [PKDB], http://pkdb.mayo.edu).

Pathogenic mutation conformation

Mutations identified in the current study were categorized into three classes: definite pathogenic mutation, probable pathogenic mutations and indeterminate mutation. The definite pathogenic mutations referred to nonsense mutations, frameshift deletions, insertions or indels, typical splicing mutations, or in-frame changes of ≥ 5 amino acids. According to a previous study, the probably pathogenic mutations referred primarily to missense mutations with a combined a core of ≥ 14 (see below)(2). The indeterminate mutations referred to the remaining mutations.

The potential pathogenicity of the identified missense variants was evaluated by combining different methods: the Grantham matrix scoring system Align Grantham Variation Grantham Deviation (Align-GVGD) (http:// agvgd.iarc.fr/)(3), PolyPhen-2 (Polymorphism Phenotyping v2; http://genetics.bwh.harvard.edu/pph/)(4), Sorting Intolerant from Tolerant (SIFT) (http://sift.jcvi.org/)(5), and Mutation Taster(6). Based on the results of these analyses, each of the identified missense variations was scored as follows: (1) A Grantham distance <60 corresponded to a score of 0, and a Grantham distance > 60 corresponded to a score of 2; (2) using A-GVGD, a score from 0 to 6 was assigned for C0 to C65; (3) a PolyPhen score of 0, 2, or 4 was assigned to benign, possibly damaging or probably damaging variations, respectively; (4) using SIFT and Mutation Taster, a score of 0 or 4 was assigned to tolerated or non-tolerated, disease-causing changes, respectively.

Potential splice-site effects were evaluated using Splice Site Prediction based on Neural

Network and Human Splice Finder software. Variations that were predicted to affect splicing by both methods were considered to be "definite pathogenic mutations". Variations that were predicted to affect splicing by only one of these software applications were considered to be a "probably pathogenic mutation". A variation that was predicted to have no effect on splicing by both software applications was considered to be "indeterminate mutation".

Reference

1. den Dunnen, JT, Antonarakis, SE: Mutation nomenclature extensions and suggestions to describe complex mutations: a discussion. *Hum Mutat* 15**:** 7-12, 2000
2. Audrezet, MP, Cornec-Le Gall, E, Chen, JM, Redon, S, Quere, I, Creff, J, Benech, C, Maestri, S, Le Meur, Y, Ferec, C: Autosomal dominant polycystic kidney disease: comprehensive mutation analysis of PKD1 and PKD2 in 700 unrelated patients. *Hum Mutat* 33**:** 1239-1250, 2012
3. Mathe, E, Olivier, M, Kato, S, Ishioka, C, Hainaut, P, Tavtigian, SV: Computational approaches for predicting the biological effect of p53 missense mutations: a comparison of three sequence analysis based methods. *Nucleic Acids Res* 34**:** 1317-1325, 2006
4. Adzhubei, IA, Schmidt, S, Peshkin, L, Ramensky, VE, Gerasimova, A, Bork, P, Kondrashov, AS, Sunyaev, SR: A method and server for predicting damaging missense mutations. *Nat Methods* 7**:** 248-249, 2010
5. Ng, PC, Henikoff, S: Predicting deleterious amino acid substitutions. *Genome Res* 11**:** 863-874, 2001
6. Schwarz, JM, Rodelsperger, C, Schuelke, M, Seelow, D: MutationTaster evaluates disease-causing potential of sequence alterations. *Nat Methods* 7**:** 575-576, 2010

Supplementary Tables

Supplementary Table 1. Average sequencing quality control and coverage statistics of targeted sequencing

Exome Capture Statistics	Average	SD
Target region (bp)(1)	12831.00	0.000
Raw reads	1158866.00	181017.199
Raw data yield (Mb)	104.30	16.371
Reads mapped to genome	368578.80	43045.447
Reads mapped to target region(2)	26046.40	3235.389
Data mapped to target region (Mb)	1.6930	0.21045
Mean depth of target region(X)	131.9000	16.39786
Coverage of target region (%)	98.9920	0.36408
Average read length (bp)	89.894	0.0052
Rate of nucleotide mismatch (%)	.3290	0.01197
Fraction of target covered >=4X (%)	98.2850	0.25136
Fraction of target covered >=10X (%)	97.1650	0.44373
Fraction of target covered >=20X (%)	94.8980	0.48207
Capture specificity (%)(3)	9.3060	0.43095
Reads mapped to flanking region(4)	18045.30	2219.362
Mean depth of flanking region(X)	116.2910	14.38044
Coverage of flanking region (%)	97.9120	0.58596
Fraction of flanking covered >=4X (%)	95.9510	0.47400
Fraction of flanking covered >=10X (%)	93.6750	0.47924
Fraction of flanking covered >=20X (%)	91.9120	0.35131
Fraction of unique mapped bases on or near target (%)	14.5700	0.65955
Duplication rate (%)(5)	8.0550	0.20018
GC rate (%)	52.4530	0.69527

Supplementary Table 2. Mutations predicted in PKD1/PKD2

no.	Gene Name	Exon/Intron	Type	Codons	cDNA change	Substitution	Share Number	Known/novel	Reference
Definite pathogenic mutation									
1	PKD2	14	nonsense	CGA2533TGA	c.2533C>T	R845*	2	Novel	
2	PKD2	4	nonsense	CGA1081TGA	c.1081C>T	R361*	2	Novel	
3	PKD2	4	nonsense	CGA958TGA	c.958C>T	R320*	1	Novel	
4	PKD1	6	nonsense	CAG1297TAG	c.1297C>T	Q433*	1	Novel	
5	PKD1	7	nonsense	TGG1548TGA	c.1548G>A	W516*	2	Novel	
6	PKD1	8	frameshift		c.1686_1687insTG		1	Novel	
7	PKD1	15	frameshift		c.3300_3301insT		1	Novel	
8	PKD1	15	nonsense	GAG3334TAG	c.3334G>T	E1112*	1	Novel	
9	PKD1	15	frameshift		c.4382_4383insC		1	Novel	
10	PKD1	15	frameshift		c.4493_4494insA		1	Novel	
11	PKD1	15	nonsense	TCG4712TAG	c.4712C>A	S1571*	3	Novel	
12	PKD1	15	nonsense	TGC5940TGA	c.5940C>A	C1980*	1	Novel	
13	PKD1	15	nonsense	CAG6307TAG	c.6307C>T	Q2103*	1	Known	10
14	PKD1	15	nonsense	CAG6472TAG	c.6472C>T	Q2158*	2	Known	30
15	PKD1	15	Indel		c.6671_6672delGGCAGCGCCAGCCGC		1	Novel	
16	PKD1	15	nonsense	CAG6736TAG	c.6736C>T	Q2246*	1	Known	Athena Diagnositics (Unpublished)

17	PKD1	18	nonsense	CGA7288TGA	c.7288C>T	R2430*	1	Known	10,18
18	PKD1	21	nonsense	CAG7987TAG	c.7987C>T	Q2663*	1	Known	10
19	PKD1	23	Indel				1	Novel	
20	PKD1	24	nonsense	CAG8905TAG	c.8905C>T	Q2969*	2	known	10
21	PKD1	24	frameshift		c.8692_8693insAACGGAGTT		1	Novel	
22	PKD1	32	frameshift				1	Novel	
23	PKD1	33	nonsense	CAG10321TAG	c.10321C>T	Q3441*	1	Novel	
24	PKD1	34	nonsense	CAG10420TAG	c.10420C>T	Q3474*	1	known	30
25	PKD1	35	nonsense	GAA10585TAA	c.10585G>T	E3529*	1	Novel	
26	PKD1	36	frameshift		c.10216_10217insA		1	Novel	
27	PKD1	40	frameshift		c.11331_11332delG		1	Novel	
28	PKD1	Intron 41-42	spliceAcceptorSite	-	-	-	1	Novel	
29	PKD1	43	frameshift		c.11767_11768insCCAA		1	Novel	
30	PKD1	43	nonsense	CAG11935TAG	c.11935C>T	Q3979*	3	known	10
31	PKD1	44	nonsense	CAG12124TAG	c.12124C>T	Q4042*	1	known	10
32	PKD1	Intron 44-45	spliceDonorSite	-	-	-	1	Novel	
33	PKD1	46	nonsense	CAG12577TAG	c.12577C>T	Q4193*	1	Novel	
Probably pathogenic mutation									
1	PKD2	Intron 8-9	spliceDonorSite	-	-	-	5	Novel	
2	PKD2	8	missense	TCG1760TTG	c.1760C>T	S587L	1	Novel	
3	PKD2	4	missense	CGG965CCG	c.965G>C	R322P	2	Novel	

4	PKD1	6	missense	GCG1295GTG	c.1295C>T	A432V	1	Known	9
5	PKD1	Intron 8-9	missense	CGG1550CAG	c.1550G>A	R517Q	1	Novel	
6	PKD1	15	missense	ACG3344ATG	c.3344C>T	T1115M	1	Novel	
7	PKD1	15	missense	GGG3490AGG	c.3490G>A	G1164R	2	Novel	
8	PKD1	15	missense	GGG3955AGG	c.3955G>A	G1319R	1	Novel	
9	PKD1	15	missense	CGC4090TGC	c.4090C>T	R1364C	1	Novel	
10	PKD1	15	missense	GGC5855GAC	c.5855G>A	G1952D	1	Known	15
11	PKD1	15	missense	TCC6842TTC	c.6842C>T	S2281F	1	Novel	
12	PKD1	15	missense	GAC6868TAC	c.6868G>T	D2290Y	1	Novel	
13	PKD1	17	missense	TCC7100TTC	c.7100C>T	S2367F	2	Novel	
14	PKD1	17	missense	GCC7130GTC	c.7130C>T	A2377V	1	Novel	
15	PKD1	18	missense	TGC7483CGC	c.7483T>C	C2495R	1	Known	10
16	PKD1	23	missense	ATG8279AAG	c.8279T>A	M2760K	1	Novel	
17	PKD1	23	missense	GAG8311AAG	c.8311G>A	E2771K	1	Known	9
18	PKD1	23	missense	GGC8353CGC	c.8353G>C	G2785R	2	Novel	
19	PKD1	23	missense	TTC8558TCC	c.8558T>C	F2853S	2	known	14
20	PKD1	23	missense	ACG8780ATG	c.8780C>T	T2927M	1	known	Athena Diagnositics (Unpublished)
21	PKD1	Intron 24-25	spliceDonorSite	-	-	-	1	Novel	
22	PKD1	25	missense	ACA9080AGA	c.9080C>G	T3027R	1	Novel	
23	PKD1	27	missense	CGA9548CAA	c.9548G>A	R3183Q	1	Known	18,25
24	PKD1	28	missense	CCT9578CTT	c.9578C>T	P3193L	1	Known	10
25	PKD1	29	missense	CGT9763TGT	c.9763C>T	R3255C	2	Novel	
26	PKD1	Intron 36-37	spliceSite				1	Novel	
27	PKD1	Intron 37-38	spliceDonorSite	-	-	-	1	Novel	

28	PKD1	39	missense	CGG11248TGG	c.11248C>T	R3750W	4	Novel	
29	PKD1	39	missense	CGG11257TGG	c.11257C>T	R3753W	2	known	9,10
30	PKD1	Intron 42-43	spliceSite	-	-	-	1	Novel	
31	PKD1	46	missense	AAA12455ACA	c.12455A>C	K4152T	1	known	Athena Diagnositics (Unpublished)
32	PKD1	46	missense	TCC12569TTC	c.12569C>T	S4190F	2	known	30
33	PKD1	46	3-UTR				23	Novel	
34	PKD1	46	3-UTR				2	Novel	

Indeterminate mutation

1	PKD2	5	missense	AGA1190AAA	c.1190G>A	R397K	2	Novel	
2	PKD1	10	none	GCC1910GTC	c.1910C>T	A637V	1	Known	31
3	PKD1	10	missense	GTT2068ATT	c.2068G>A	V690I	1	Novel	
4	PKD1	14	missense	ATG3276ATC	c.3276G>C	M1092I	2	Novel	
5	PKD1	15	missense	TCG3548TGG	c.3548C>G	S1183W	1	Novel	
6	PKD1	15	missense	GTG3565ATG	c.3565G>A	V1189M	1	Novel	
7	PKD1	15	missense	GAT3613CAT	c.3613G>C	D1205H	1	Novel	
8	PKD1	15	missense	GTG3865ATG	c.3865G>A	V1289M	1	Novel	
9	PKD1	15	missense	CTG3868GTG	c.3868C>G	L1290V	1	known	25
10	PKD1	15	missense	ACC4336CCC	c.4336A>C	T1446P	5	Novel	
11	PKD1	15	missense	ACC4541ATC	c.4541C>T	T1514I	1	Novel	
12	PKD1	15	missense	GTG4810ATG	c.4810G>A	V1604M	1	Known	25
13	PKD1	15	missense	TCC5176ACC	c.5176T>A	S1726T	1	Novel	
14	PKD1	15	missense	GGC5770AGC	c.5770G>A	G1924S	2	Novel	
15	PKD1	15	missense	CAG6032CGG	c.6032A>G	Q2011R	1	Novel	
16	PKD1	15	missense	CGG6440CAG	c.6440G>A	R2147Q	1	Novel	

17	PKD1	15	missense	GCG6665GTG	c.6665C>T	A2222V	4	known	Athena Diagnositics (Unpublished)
18	PKD1	16	missense	GCG6935GTG	c.6935C>T	A2312V	1	known	Athena Diagnositics (Unpublished)
19	PKD1	Intron 16-17	spliceAcceptorSite	-	-	-	1	Novel	
20	PKD1	Intron 16-17	spliceSite	-	-	-	2	Novel	
21	PKD1	17	missense	AGC7190AAC	c.7190G>A	S2397N	1	Known	Athena Diagnositics (Unpublished)
22	PKD1	22	missense	CAC8067CAG	c.8067C>G	H2689Q	1	Novel	
23	PKD1	22	missense	CTC8087CGC	c.8087T>G	L2696R	2	Known	25
24	PKD1	Intron 22-23	spliceDonorSite	-	-	-	1	Novel	
25	PKD1	23	missense	GAG8224AAG	c.8224G>A	E2742K	1	Known	18
26	PKD1	23	missense	CGC8300CAC	c.8300G>A	R2767H	3	Known	Athena Diagnositics (Unpublished)
27	PKD1	23	missense	GTG8302TTG	c.8302G>T	V2768L	1	Novel	
28	PKD1	23	missense	GCC8671ACC	c.8671G>A	A2891T	2	Novel	
29	PKD1	26	missense	GTG9256ATG	c.9256G>A	V3086M	1	Novel	
30	PKD1	26	missense	TTC9331ATC	c.9331T>A	F3111I	1	Novel	
31	PKD1	Intron 28-29	spliceSite	-	-	-	1	Novel	
32	PKD1	29	missense	AAC9884AGC	c.9884A>G	N3295S	1	known	25
33	PKD1	31	missense	GAC10102AAC	c.10102G>A	D3368N	2	known	24
34	PKD1	Intron 34-35	spliceDonorSite	-	-	-	2	Novel	

35	PKD1	Intron 34-35	spliceDonorSite	-	-	-	2	Novel	
36	PKD1	36	missense	CTC10801TTC	c.10801C>T	L3601F	1	Novel	
37	PKD1	Intron 38-39	spliceSite	-	-	-	42	Novel	
38	PKD1	40	missense	ACC11333AAC	c.11333C>A	T3778N	1	known	25
39	PKD1	Intron 14-42	spliceSite	-	-	-	1	Novel	
40	PKD1	42	missense	CGC11567CAC	c.11567G>A	R3856H	2	Novel	
41	PKD1	Intron 42-43	spliceSite	-	-	-	1	Novel	

Supplementary Table 3. Mutation detection and prediction in PKD1/PKD2

	PKD1	PKD2
Nonsense	17	3
Frameshift	9	3
Indel	2	
Missense	55	
SpliceSite	16	1
3’-UTR	2	
**Total**	**101**	**7**
Definite pathogenic mutation	30	3
Probably pathogenic mutation	31	3
Indeterminate mutation	40	1
**Total**	**101**	**7**

Supplementary Table 4. Influence of PKD2 mutation on clinical phenotype

**Characteristic**	**PKD1 mutation (Indeterminate type) (n=49)**	**PKD1 mutation(Indeterminate type)+PKD2 mutation (n=6)**	***P* Value**
Sex (male/female)	29/20	4/2	0.767
Age (yr)	44.98±14.06	50.33±11.81	0.376
Age at diagnosis (yr)	37.14±11.22	38.75±0.50	0.779
Clear family history	25 (52.1%)	5 (83.3%)	0.204
Polycystic liver	16(33.3%)	5 (83.3%)	0.026
hypertension	27(57.4%)	4 (66.7%)	0.686
Urologic complication	28(58.3%)	5 (83.3%)	0.384
Born as the first child	40(83.3%0	4 (66.7%)	0.588
BMI (kg/m^2)	22.40(21.22-25.71)	25.11(17.14-27.90)	0.293
Hemoglobin (g/L)	128.87±19.75	139.80±17.11	0.253
White blood cell count (*10^9/L)	6.54±2.18	6.02±1.12	0.604
Platelet (*10^9/L)	225.48±75.07	171.00±29.18	0.123
Serum albumin (g/L)	43.63±5.42	42.52±6.74	0.686
Serum total protein (g/L)	72.34±5.85	64.40±5.34	0.008
Serum creatinine (μmol/L)	90.30(64.65-115.50)	79.10(59.85-150.90)	0.476
Serum urea nitrogen (mmol/L)	7.00(4.97-8.07)	5.45(3.33-9.74)	0.365
Serum uric acid (μmol/L)	323.35±111.34	309.60±92.10	0.794
CysC (mg/L)	0.92(0.71-1.37)	1.09(0.76-2.16)	0.505
GFR (ml/min)	79.13(55.85-108.75)	93.75(48.17-110.77)	0.986
Urine protein quantity (g/24h)	0.19(0.00-1.43)	0.13(0-1.22)	0.884
Kidney volume (mm^3)	269.61(193.17-742.87)	289.02(192.11-894.32)	0.102

BMI, Body Mass Index; eGFR, estimated Glomerular Filtration Rate

Supplementary Table 5. Influence of mutation position in targeted regions on clinical phenotype of PKD1

**Characteristic**	**Position 1 (n=15)**	**Position 2 (n=22)**	**Position 3 (n=18)**	***P* Value**
Sex (male/female)	8/7	7/15	8/10	0.412
Age (yr)	37.43±13.11	43.11±13.11	46.75±12.96	0.140
Age at diagnosis (yr)	33.00±6.48	33.31±8.93	34.00±7.10	0.949
Clear family history	8 (53.3%)	11 (50.0%)	8 (4.4%)	0.873
Polycystic liver	5(33.3%)	4(18.2%)	6(33.3%)	0.466
hypertension	7(50.0%)	10(45.5%0	12(66.7%0	0.388
Urologic complication	10(66.7%0	9(40.9%0	8(44.4%0	0.273
Born as the first child	15(100%)	18(818%)	16(88.9%)	0.219
BMI (kg/m^2)	24.36(21.36-27.63)	22.03(21.26-22.80)	22.80(20.68-24.89)	0.308
Hemoglobin (g/L)	127.67±29.97	131.08±14.02	125.20±18.42	0.774
White blood cell count (*10^9/L)	7.15±1.95	5.58±1.47	6.47±1.89	0.137
Platelet (*10^9/L)	209.67±82.48	190.17±48.06	239.00±103.71	0.381
Serum albumin (g/L)	45.80±3.42	43.36±4.92	44.13±5.46	0.576
Serum total protein (g/L)	70.97±6.01	70.45±5.69	72.89±7.34	0.720
Serum creatinine (μmol/L)	92.00(66.20-201.05)	80.00(56.83-129.95)	110.50(75.63-235.20)	0.253
Serum urea nitrogen (mmol/L)	8.64(5.39-9.88)	7.20(4.58-9.23)	7.47(6.75-14.44)	0593
Serum uric acid (μmol/L)	350.36±106.99	334.68±121.22	318.88±133.52	0.815
CysC (mg/L)	1.32(0.89-2.00)	0.86(0.65-1.56)	1.11(0.83-2.65)	0.520
GFR (ml/min)	90.58(23.31-106.48)	89.21(44.16-116.68)	50.49(22.39-109.68)	0.329
Urine protein quantity (g/24h)	0.10(0.05-0.45)	0.14(0.02-1.18)	0.58(0.05-1.31)	0.691
Kidney volume (mm^3)	563.11(112.44-936.54)	277.17(233.78-981.66)	326.71(178.22-853.26)	0.911

BMI, Body Mass Index; eGFR, estimated Glomerular Filtration Rate

Supplementary Table 6. Influence of mutation pathogenic type on clinical phenotype of PKD1 (patients with single mutation)

Characteristic	Definite pathogenic (n=15)	Probably pathogenic (n=8)	Indeterminate (n=32)	*P* Value
Sex (male/female)	5/10	2/7	17/14	0.040
Age (yr)	42.17±8.87	46.60±14.26	42.25±14.18	0.780
Age at diagnosis (yr)	34.67±5.54	30.00±9.17	33.46±8.03	0.656
Clear family history	7 (46.7%)	1 (12.5%)	19 (59.4%0	0.059
Polycystic liver	2(13.3%)	2(25.0%)	11(27.3%)	0.316
hypertension	5(33.3%)	3(37.5%0	21(67.7%)	0.055
Urologic complication	6(40.0%)	2(25.0%)	19(59.4%)	0.157
Born as the first child	14(93.3%)	8(100%)	27(84.4%)	0.370
BMI (kg/m^2)	22.59(20.50-24.85)	22.14(20.28-24.11)	22.21(21.39-25.31)	0.791
Hemoglobin (g/L)	129.25±22.55	129.67±12.22	127.71±19.43	0.975
White blood cell count ($*10^9/L$)	5.78±1.60	5.65±0.67	6.61±1.98	0.478
Platelet ($*10^9/L$)	171.14±52.50	173.33±44.99	230.10±84.26	0.159
Serum albumin (g/L)	44.02±3.05	45.60±7.25	44.50±5.37	0.940
Serum total protein (g/L)	68.20±3.88	70.65±10.39	72.92±6.31	0.065
Serum creatinine (μmol/L)	104.20(73.03-205.80)	136.40(103.00-229.80)	80.00(54.05-112.70) [ab]	0.007
Serum urea nitrogen (mmol/L)	6.81(5.20-8.41)	8.14(6.30-10.00)	6.40(4.68-7.65)[a]	0.008
Serum uric acid (μmol/L)	333.12±118.70	330.54±136.00	327.49±123.09	0.992
CysC (mg/L)	1.12(0.79-2.18)	1.25(0.61-1.56)	0.87(0.66-1.44)[a]	0.017
Urine protein quantity (g/24h)	0.19(0.02-0.70)	0.13(0.06-0.20)	0.14(0-1.12)	0.714
eGFR (ml/min)	62.00(26.91-91.91)	40.24(25.30-51.22)	90.58(49.75-117.03)[ab]	0.022
Kidney volume (mm^3)	829.80(228.36-1285.42)	557.41(226.76-888.06)	276.11(198.94-482.11)	0.265

BMI, Body Mass Index; eGFR, estimated Glomerular Filtration Rate

[a] $P<0.05$ compared with group with definite pathogenic mutation

[b] $P<0.05$ compared with group with probably pathogenic mutation

Supplementary Table 7. Influence of mutation pathogenic type on clinical phenotype of PKD1 (patients with single mutation)

Characteristic	Pathogenic (n=23)	Indeterminate (n=32)	Without variants (n=21)	*P* Value
Sex (male/female)	6/17	17/14	11/10	0.098
Age (yr)	43.47±10.45	42.25±14.18	40.24±11.58	0.723
Age at diagnosis (yr)	33.50±6.49	33.46±8.03	32.55±12.54	0.956
Clear family history	8 (34.8%)	19 (59.4%)	8 (38.1%0	0.136
Polycystic liver	4(17.4%0	11(27.3%)	5(23.8%)	0.353
hypertension	8(34.8%)	21(67.7%)	1(52.4%)	0.056
Urologic complication	8(34.8%)	19(59.4%)	9(42.9%)	0.175
Born as the first child	22(95.7%)	27(84.4%)	18(85.7%)	0.407
BMI (kg/m^2)	22.54(20.52-24.93)	22.21(21.39-25.31)	23.09(20.38-24.85)	0.772
Hemoglobin (g/L)	129.36±19.64	127.71±19.43	135.09±10.94	0.536
White blood cell count (*10^9/L)	5.74±1.35	6.61±1.98	5.93±1.05	0.321
Platelet (*10^9/L)	171.80±47.84	230.10±84.26	192.20±59.05	0.096
Serum albumin (g/L)	44.18±3.26	44.50±5.37	46.42±5.28	0.551
Serum total protein (g/L)	68.20±3.88	72.92±6.31	74.66±3.65 [a]	0.043
Serum creatinine (μmol/L)	173.88(81.40-239.35)	80.00(54.05-112.70)	57.55(53.50-101.55) [a]	0.001
Serum urea nitrogen (mmol/L)	8.96(7.36-14.44)	6.40(4.68-7.65)[a]	5.03(3.82-6.99) [a]	0.002
Serum uric acid (μmol/L)	332.12±121.72	327.49±123.09	303.17±133.22	0.798
CysC (mg/L)	1.56(0.88-2.66)	0.87(0.66-1.44)[a]	0.66(0.53-1.23) [a]	0.011
GFR (ml/min)	35.08(20.25-103.87)	90.58(49.75-117.03)	102.72(72.80-126.90)[a]	0.005
Urine protein quantity (g/24h)	0.19(0.07-0.70)	0.14(0-1.12)	0.15(0.00-0.60)	0.619
Kidney volume (mm^3)	864.11(592.92-1677.72)	276.11(198.94-482.11)	188.19(113.68-353.69) [a]	0.102

BMI, Body Mass Index; eGFR, estimated Glomerular Filtration Rate

[a] P<0.05 compared with group with pathogenic mutation

Supplementary Figure 1. Schematic diagram for the Next Generation Sequencing

A custom capture array (NimbleGen, Roche) was designed to capture the exons, splice sites and the immediately adjacent intron sequences of targeted genes (PKD1 and PKD2). The qualified genomic DNA sample was then fragmented using AFA (Adaptive Focused Acoustics™) technology on Covaris S220 Focused-ultrasonicators, and the sizes of the library fragments were primarily distributed between 250 bp and 300 bp. Adapters were then ligated to both ends of the resulting fragments. Extracted DNA was then amplified by ligation-mediated PCR (LM-PCR), purified, and hybridized to the Nimblegen SeqCap EZ Library for enrichment; non-hybridized fragments were then washed out. Both non-captured and captured LM-PCR products were subjected to quantitative PCR to estimate the magnitude of enrichment. Each captured library was then loaded on a Hiseq2000 platform, and we performed high-throughput sequencing for each captured library to ensure that each sample met the desired average sequencing depth. Raw image files were processed using the Illumina basecalling Software 1.7 for base-calling with default parameters, and the sequences of each individual were generated as 90 bp pair-end reads. SOAPaligner/SOAP2 was used to map reads onto the reference, whereas BWA was used to map reads to the reference when analysing indels. The information of BWA can be found at http://bio-bwa.sourceforge.net/. The variations identified with NGS were validated by using classic Sanger sequencing. The standard nomenclature recommended by HGVS (http://www.hgvs.org/mutnomen/) was used to name the variations.

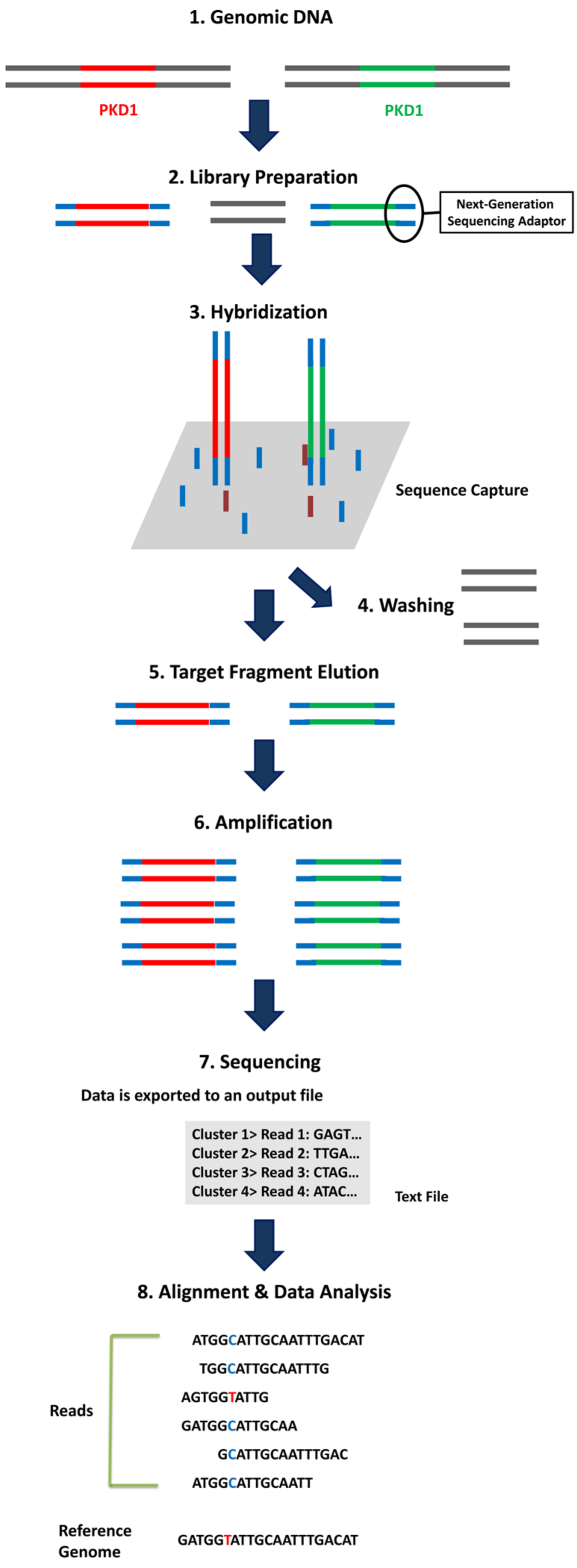

Supplementary Figure 1. Schematic diagram for the Next Generation Sequencing
